# Cathepsin B Is Upregulated and Mediates ECM Degradation in Colon Adenocarcinoma HT29 Cells Overexpressing Snail

**DOI:** 10.3390/cells8030203

**Published:** 2019-02-27

**Authors:** Jakub Kryczka, Izabela Papiewska-Pajak, M. Anna Kowalska, Joanna Boncela

**Affiliations:** Institute of Medical Biology, Polish Academy of Sciences, 93-232 Lodz, Poland; jkryczka@cbm.pan.pl (J.K.); ipapiewska-pajak@cbm.pan.pl (I.P.-P.); mkowalska@cbm.pan.pl (M.A.K.)

**Keywords:** EMT, Snail, mesenchymal type of migration, proteases

## Abstract

During tumor development and ongoing metastasis the acquisition of mesenchymal cell traits by epithelial carcinoma cells is achieved through a programmed phenotypic shift called the epithelial-to-mesenchymal transition, EMT. EMT contributes to increased cancer cell motility and invasiveness mainly through invadosomes, the adhesion structures that accompany the mesenchymal migration. The invadosomes and their associated proteases restrict protease activity to areas of the cell in direct contact with the ECM, thus precisely controlling cell invasion. Our data prove that Snail-overexpressing HT-29 cells that imitate the phenotype of colon cancer cells in the early stage of the EMT showed an increase in the expression and pericellular activity of cathepsin B. It appears that the pericellular localization of cathepsin B, also observed in colon and rectum adenocarcinoma tissue samples, plays a key role in its function.

## 1. Introduction

Cancer progression is mainly characterized by rapid increases in tumor mass and volume (in solid tumors), increased invasiveness, and overall decreases in patient survival rates [1]. The propensity of cancer cells to disseminate from primary tumors has been known for over a century. The underlying molecular mechanisms of this phenomenon are rather complex and remain largely unexplored [2]. Regardless, cancer cells undergo sequential steps to form metastases. To undergo these steps, cancer cells first have to acquire a migratory phenotype and disrupt the tissue architecture, which requires modulation of cell-matrix and cell-cell contacts [3,4,5]. Therefore, molecules involved in cancer cell migration could be potential targets for anti-metastatic therapy. The plasticity of cancer cells is governed by the epithelial-to-mesenchymal transition (EMT) [6]. The EMT is a mechanism that, under physiological conditions, is involved in wound healing and organism development [7]. During the EMT, cells lose their origin markers, polarity, and cell-cell connections and gain mesenchymal-like pro-migratory phenotypes that enable them to cross anatomical boundaries such as the extracellular matrix (ECM) or the basement membrane [7,8]. In tumors EMT contributes to increased cancer cell motility and invasiveness [9,10]. The mesenchymal mode (type) of migration is strongly dependent on GTPase activity, proteolytic degradation of ECM components and adhesion via integrins [11]. The “path-generating” ability of cells during the EMT is mainly mediated by invadosomes, which are actin-rich, highly dynamic adhesion structures. The discovery of invadosomes on the surface of invasive and metastatic cancer cells has provided important new insight into the cellular and molecular basis of malignant progression. Recent studies suggest a role for invadosomes and their associated proteases in localized matrix degradation during cell invasion; in other words, invadosomes restrict protease activity to areas of the cell in direct contact with the ECM, thus precisely controlling cell invasion in vivo [12,13,14]. The matrix-degrading abilities of invadosomes are mediated mainly by matrix metalloproteinases (MMPs). MMP-14, a membrane-associated enzyme that has the ability to activate other MMPs, such as MMP-2, from their inactive isoforms as well as to degrade collagen, gelatine, fibronectin and laminin, is consistently present in invadosomes [15,16,17,18]. Several other MMPs, such as MMP-2 and MMP-9, also contribute to ECM degradation by invadosomes [16,18,19]. However, even though MMPs have been well known to mediate metastasis and induce tumor progression since the early 1990s, clinical trials targeting MMPs have resulted in failure, as a broad range of MMP inhibitors cause severe side effects [20,21]. Currently, new insight into the biological role of MMPs and their complex substrate network (ECM components, cell surface receptors, cell-cell contact proteins, chemokines and signaling molecules) supports the assumption that other proteases may be more effective as anti-migratory and thus anti-metastatic targets [22]. Cathepsins were first linked to cancer many years ago [23]. It is now clear that cathepsins play a prominent role in both tumor progression and metastasis, which is supported by numerous clinical reports and results from experimental mouse models of cancer, although the precise mechanisms by which cathepsins exert their effect are still under extensive study. Murine knock-out models have indicated that the absence of cathepsin B, S or L impairs tumor invasion. Furthermore, mutation of cathepsin B or S impairs tumor formation and angiogenesis, whereas knock-out of cathepsin B or L decreases cell proliferation and tumor growth [24]. Cathepsin B-producing macrophages are associated with increased local tumor recurrence during perineural invasion (PNI) [25]. These results showed that each cathepsin member exerts different functions in the process of tumor metastasis. This fact will enable the development of individualized treatments for cancer patients based on the type and progression of cancer. Cathepsin inhibitors are already being tested in clinical trials and hold promise for combined cancer therapies. Specifically, the monoclonal antibody Fsn0503, which targets cathepsin S, and the small-molecule cathepsin S inhibitor Z-FL-COCHO were shown to suppress invasion in colorectal, prostate and breast cancer cell lines and to suppress tumor growth in vivo in a colorectal tumor xenograft model [21,26]. Cathepsin activity in cancer cells is also being used as a target to release the cytotoxic payload of an antibody-drug conjugate (ADC), brentuximab vedotin, via a protease-cleavable dipeptide valine-citrulline (VC) linker in anti-lymphoma therapy [27]. The cathepsin inhibitors CA-074, AMS-36 and LHVS significantly decrease the ability of macrophages to invasion [28], which confirms that cathepsins are present in invadosome structures and are involved in cell motility [22]. Of note, the cysteine cathepsins B, X, S, H, and L were found to accumulate at the degradative tips of invadosome protrusions in v-Src fibroblasts, human blood macrophages, the human monocytic cell line U-937, cardiomyocytes and osteoclasts [28,29,30]. Lysosomal cathepsins are delivered and released to formed invadosomes protrusions by lysosomal fusion [31]. Metabolism in cancer cells that preferentially use a glycolytic pathway leads to an increased production of lactic acid and results in a decreased pH of the microenvironment, which enhances cathepsin activity [31,32]. Furthermore, invadosomes are enriched in glycolytic enzymes that in turn increase proton efflux to the microenvironment, and local acidification increases the activity of cysteine cathepsins [33]. Even though cathepsins have a wide range of impacts on cancer metastasis, the primary targets for anti-migratory and anti-ECM-remodeling therapy are still MMPs. Our previous studies on HT-29 colon carcinoma cells with Snail overexpression, which mimic colon cancer cells at the early stage of the EMT, proved that HT-29/Snail cells have a higher migratory rate than normal HT-29 cells; however, no upregulation or enhanced activity of MMP-2 or MMP-14 was observed [9,34]. That finding led us to assume that other proteases, e.g., cathepsins, might be responsible for the increased migration rates and ECM-degrading ability of colon cancer cells.

## 2. Methods

### 2.1. Cell Culture and Reagents

Colon cancer cell line HT-29 was obtained from American Type Culture Collection (Manassas, VA, USA) and cultured in McCoy’s 5A medium (Life Technologies, Waltham, MA, USA), supplemented with 10% FBS (Life Technologies, Waltham, MA, USA) and antibiotics - streptomycin and penicillin (Sigma-Aldrich, St. Louis, MO, USA), primocin (Invivogen, San Diego, CA, USA) in a 90–95% humidified atmosphere of 5% CO2. The cells were periodically tested for mycoplasma every 4 weeks using the PlasmoTest (Invivogen, San Diego, CA, USA). The following inhibitors were used: ARP101 ((*R*)-N-Hydroxy-2-(*N*-isopropoxybiphenyl-4-ylsulfonamido)-3-methylbutanamide, Tocris Bioscience, Bristol, UK), MMP-2 inhibitor I (Oleoyl-*N*-hydroxylamide; *N*-Hydroxy-9-octadecenamide, Snata Cruz Biotechnology, Santa Cruz, CA, USA), Furin inhibitor I (N2-(1-oxodecyl)-l-arginyl-l-valyl-*N*-[(1*S*)-4-[(aminoiminomethyl)amino]-1-(2-chloroacetyl)butyl]-l-lysinamide, Cayman, Chemical Ann Arbor, MA, USA), Cathepsin B inhibitor CA-074Me (*N*-[[(2S,3S)-3-[(propylamino)carbonyl]-2-oxiranyl]carbonyl]-*L*-isoleucyl-L-proline, methyl ester; Cayman, Chemical Ann Arbor, MA, USA), BD Matrigel Matrix Growth Factor Reduced, Phenol Red-Free #356231 (Becton Dickinson, Franklin Lakes, NJ, USA).

### 2.2. Western Immunoblotting 

Proteins isolated from HT-29 cells were extracted with NP-40 lysis buffer (50 mM Tris, pH 8.0, containing 1% Nonidet-Igepal, 150 mM NaCl, 5 mM EDTA) with the Halt protease inhibitor cocktail (Thermo Scientific, Waltham, MA, USA), and the soluble protein fraction was collected through centrifugation. The protein concentrations in the cell lysates were measured with the BCA method (Pierce/Thermo Scientific, Waltham, MA, USA) and were equalized between samples. The protein extracts were subjected to SDS-PAGE analysis and were electro transferred onto PVDF or nitrocellulose membranes (BioRad, Hercules, CA, USA) followed by immunodetection through rabbit anti-cortactin, rabbit anti-Nck1/2 and mouse anti-Grb2 (Santa Cruz Biotechnology, Dallas, TX, USA), rabbit anti human CTSB #C17120557 (US Biological Salem, MA, USA), and rabbit anti human CTSC ARP60116_P050 (Aviva Systems Biology, San Diego, CA, USA). Proteome Profiler Human Protease Array Kit (R&D System, ARY021) was used according the manufacturer’s procedure for the screening of changes in proteases expression level in the cell culture supernatants and lysates. The control-mouse rabbit anti-β-actin antibody conjugated with HRP was obtained from Abcam (Cambridge, UK) and used as a loading control. Detection was performed using secondary HRP-conjugated antibodies (Santa Cruz Biotechnology, Dallas, TX, USA) followed by incubation with an enhanced chemiluminescence kit (Thermo Scientific, Waltham, MA, USA) and development with Kodak BioMax Light Film (Eastman Kodak, Rochester, NY, USA).

### 2.3. Fluorescent Dequenching (DQ) Gelatine Assay

The surface of 24-well plates was coated with 250 µL 0.1 mg/mL DQ gelatine (Life Technologies, Waltham, MA, USA) for overnight at 4 °C and then washed 3× with PBS. 1 × 10^5^ cells/well were added for 24 h to earlier prepared DQ gelatine-coated dishes in full medium supplemented with or without MMP-2 inhibitor ARP101, Furin inhibitor I or Cathepsin B inhibitor, CA-074ME. FITC fluorescence generated by the cleavage of DQ gelatine was measured using a Thermo Labsystem Fluoroscan Ascent microplate reader fitted with FITC excitation and emission filters. Data are presented as the percent of increase above background fluorescence (100%) observed in control HT-29. To visualize gelatine degradation, 24-well plates (250 µL/well) or chamber slides (100 µL) Lab-Tek™ (Nunc™ Lab-Tek™ Chamber Slide™ SystemThermo Scientific, Life Technologies, Waltham, MA, USA) were coated with DQ gelatine and cells were added as described above. Next, FITC fluorescence generated by the cleavage of DQ gelatine was visualized by fluorescent or confocal microscopy as described in Microscopy section. Since ARP101 was reported to induce autophagy and autophagy-associated cell death in several cancer cell lines [35], we determined viability of HT-29 Snail 8 and control cells in the presence of this inhibitor. No statistically significant changes in the % of live cells in cells treated for 24 h with 10 µM ARP101 were found compared to non-treated cells (data not shown)

### 2.4. Proteome Analysis of Cancer Tissue Samples

The analysis was performed using “The Human Protein Atlas” platform [36,37,38,39]—“Swedish-based program initiated in 2003 with the aim to map all the human proteins in cells, tissues and organs using integration of various omics technologies, including antibody-based imaging, mass spectrometry-based proteomics, transcriptomics and systems biology. All the data in the knowledge resource is open access to allow scientists both in academia and industry to freely access the data for exploration of the human proteome” (https://www.proteinatlas.org).

### 2.5. Transwell Migration Assay

HT-29 control or HT-29/Snail cells were treated with 100 µM Cathepsin B inhibitor (CA-074ME) for 24 h. Than trypsinized, washed 2× with medium and transferred (2.5 × 10^4^ cells/chamber) to upper chamber of Nunc™ Cell Culture Inserts (transwell) 8.0 μm pore diameter (#141006) covered with BD Matrigel (2 h, 0.6 mg/mL of Matrigel—75 uL) for 6 h in 0.1% BSA medium—supplemented with or without CA-074ME. Full medium in lower chamber was used as chemoattractant. Next medium and the Matrigel from the top surface of the membrane was removed, invaded cells on the bottom surface of the membrane were washed 2× with PBS, then fixed for 5 min with 96% ethanol at 4 °C. Cells were dyed at RT as follow: 6 min—hematoxylin, 1 min—1% eosin. Finally, membranes were cut out from chambers, placed on microscope glass and number of cells that migrate into the membrane was counted.

### 2.6. Viability Assay

Cell viability was calculated as the number of viable cells divided by the total number of cells automatically in a cell counter with Trypan blue staining (Invitrogen Countess; Invitrogen/Life Technologies, Waltham, MA, USA). Every sample of cell mixture was independently counted three times before each experiment using the following equation: % viable cells = [1.00 − (Number of blue cells/Number of total cells)] × 100.

### 2.7. Microscopy

The cells were grown on FITC gelatine-coated chamber slides (Life Technologies, Waltham, MA, USA) until 60–70% confluency and were subsequently incubated with Hoechst 33,342 (Molecular Probes/Life Technologies, Waltham, MA, USA) for 15 min in the incubator. The cells were washed with PBS (3×), fixed for 10 min in CellFIX^TM^ (1% formaldehyde, 0.35% methanol, 0.09% sodium azide) from BD Biosciences (cat no. 340181) for 10 min, washed with PBS (3 × 5 min) and blocked with 3% BSA/PBS at RT for 1 h. After washing with PBS, the slides were incubated with specific antibody in 1% BSA/PBS at 37 °C for 1 h. After washing with PBS, the cells were incubated with secondary antibodies conjugated with Alexa Fluor-488, Alexa Fluor-350 (Life Technologies, Waltham, MA, USA) or Texas Red (Molecular Probes/Life Technologies, Waltham, MA, USA) at RT for 1 h in the dark. The slides were washed with PBS, mounted with Mowiol (Sigma-Aldrich, St. Louis, MO, USA), and the cells were visualized under a confocal microscope (Nikon D-Eclipse C1; Nikon, Tokyo, Japan) with a 40× objective and were analyzed with EZ-C1 version 3.6 software.

### 2.8. Statistical Analysis

Data are reported as mean ± SD. Statistical comparisons between three or more sets of data were performed with a bilateral Student paired t test. Values of *p* < 0.05 are represented by one star and 0.005 by two stars. Analysis of MMP-2 and Cathepsin B inhibitors interaction was performed using Berenbaum’s equation according to the Linear Interaction Effect model and the Bliss Independence model as described by J. Foucquier and M. Guedj [40].

## 3. Results

**Figure 1:**
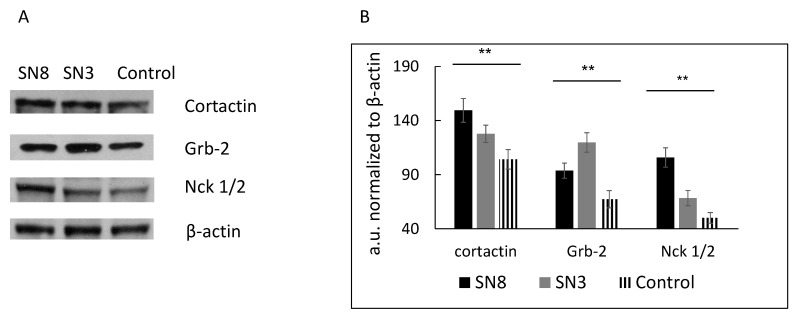
The level of invadosome related proteins in HT-29 with Snail overexpression. Protein extracts from HT-29 stably transfected with pcDNA (control) or pcDNA/Snail vector (clone 8-SN8, clone 3-SN3) were harvested and analyzed by western blot using specific antibodies as described in methods section. (**A**) Grb2, Nck1/2, and cortactin level detected by western blot and (**B**) analyzed by densitometry and ImageJ software, performed out of 5 independent western blot experiments. The level of Snail expression in HT 29 clones, SN3 and SN8 have been shown previously [9]. ** *p* > 0.005.

In our study, we used HT-29 colon cancer cells with stable overexpression of Snail, a key regulator of the EMT. The EMT has been implicated in the local dissemination of solid tumors and in subsequent metastasis. Our previous results showed that HT-29 clone 3, with moderate Snail overexpression, and HT-29 clone 8 or 17, with higher levels of Snail expression, demonstrate morphological, functional and transcriptomic profile changes, indicating EMT induction [9]. Since we observed that HT-29/Snail clones presented a significantly elevated migration rate (tested with a wound healing-like assay and by single-cell trajectory tracking), we decided to investigate invadosome formation and activity in this cellular model in the present study. First, we determined the levels of proteins involved in (i) actin rearrangement (cortactin) and (ii) invadosome formation (Grb2 and Nck1/2) using specific antibodies and the western blot technique [11]. Both Snail-positive clones, 3 and 8, presented higher expression of cortactin, Grb2 and Nck1/2 than the control cells (Figure 1A,B).

**Figure 2:**
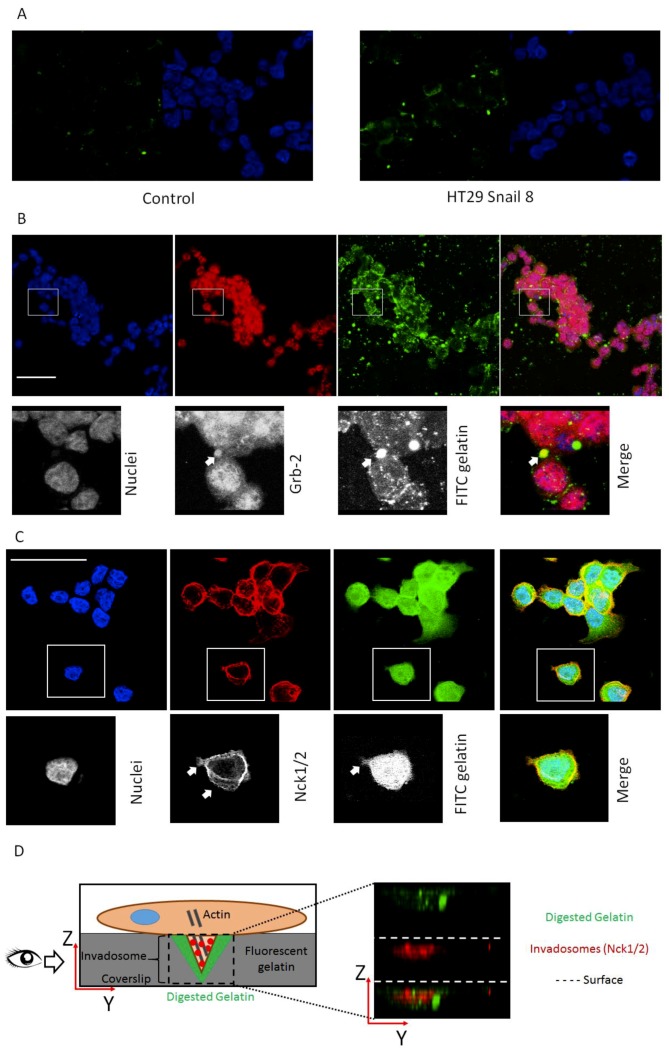
Invadosome structures formed in HT-29 cells overexpressing Snail. (**A**) The increased proteolysis of FITC-DQ gelatine (green) triggered by HT-29/Snail cells (right panel) compared to control cells (left panel) were visualized by confocal microscopy. The nuclei were stained with *Hoechst 33342* (blue). Cellular localization of invadosome related protein Grb-2 (**B**) and Nck1/2 (**C**,**D**) were visualized by confocal microscopy. (**B**) Arrows point accumulation of Grb-2 (red) with FITC-gelatine degradation spots (green). Nck1/2 co-localization with gelatine degradation was observed on both: focal-XY axis (**C**) and ventricle side of the cells—as shown on YZ axis (**D**, right panel). The cartoon visualization of Nck1/2 localization in cell (**D**, left panel). Bars indicate 50 µm.

Since cortactin, Grb-2 and Nck1/2 are highly involved in the formation of active invasive structures and are considered the core proteins in this process, we next focused on their cellular localization [41,42,43,44]. These proteins should be present in protrusions formed by the cells. Additionally, we used microscopy to examine whether Grb2 and Nck1/2 co-localize with the gelatine degradation area, which occurs in close proximity to well-formed invadosomes. For this purpose, we employed HT-29/Snail clone 8; our previous study showed that this clone was a more interesting model for early EMT studies, as the detected transcriptomic changes resembled those in response to TGFβ, an early inducer of the EMT [9]. To measure gelatinolytic activity related to the cellular invasive structure, we used in situ zymography with quenched FITC-conjugated gelatine as a substrate. Cells were seeded on chamber slides covered with quenched FITC-conjugated gelatine. After 24 h of incubation, we observed increased fluorescence in HT-29/Snail cells in areas with gelatinolytic activity derived from the cellular surface (Figure 2A). The co-localization of the Grb-2 and Nck1/2 proteins with gelatine degradation areas was visualized using confocal microscopy. The gelatinolytic areas corresponding to Grb-2 accumulation indicated clearly formed invadosomes (Figure 2B). We did not observe this effect in HT-29 control cells (Figure S1). Grb2, as an adaptor protein, is mainly localized in the cytoplasm. However, as an invadosome marker, it can be observed in cortactin- and F-actin-rich protrusions located on the ventral side of the cell, correlating with ECM degradation areas [11,45]. Nck1/2 was visualized at the cell-substratum interface (Figure 2C) and co-localized with ventral (Figure 2D) gelatine degradation areas present in the XY and XZ axes, respectively. Nck1/2 belongs to the noncatalytic region of tyrosine kinase adaptor family, whose members are involved in the propagation of extracellular signals that induce tyrosine phosphorylation and contribute to the organization of the actin cytoskeleton and the creation of invadopodia [46].

**Figure 3:**
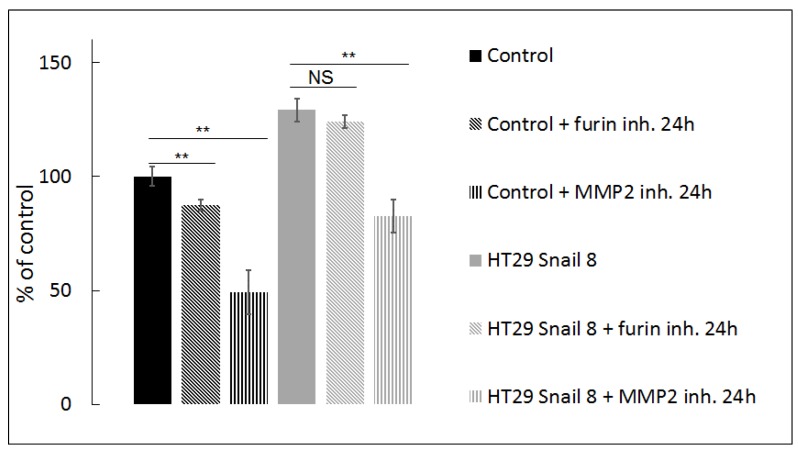
Gelatinolysis mediated by HT-29 with Snail overexpression measured by in-situ zymography. The pericellular proteolytic abilities of HT-29 cells with Snail overexpression were analyzed by measure of increase in FITC fluorescent intensity from digested DQ gelatine relativized to control cells presented as 100%. Furin inh.—25 µM Furin Inhibitor I, MMP-2 inh—10 µM ARP101. ** *p* > 0.005; NS—not statistically relevant.

Interestingly, our previous studies proved that Snail upregulation in HT-29 did not affect the expression and did not change the global activity of MMP-2, the prevalent pericellular gelatinase, in gelatine zymography tests [9,34]. Thus, to confirm our observation from confocal microscopy, in this study, we also used quenched FITC-conjugated gelatine as an MMP substrate in a DQ-gelatine degradation assay and measured fluorescence intensity as described previously [11] and in the methods section. This test is well-suited for quantifying gelatinase activity in vivo and in vitro [11,47]. Fluorescence intensity, which appears as a result of gelatine proteolysis, was significantly increased (≈130% of control levels) in HT-29/Snail cells (Figure 3, black and grey bars). These results indicate that Snail overexpression influences gelatinolytic activity in HT-29 cells and affects the cellular distribution of MMP-2 rather than its expression and global activity. Since MMP-2 activity may be increased by proteolytic cleavage mediated by MMP-14, we next tested the effect of MMP-14 inhibition in a DQ-gelatine degradation assay using a furin inhibitor (24 h incubation with 25 µM Furin Inhibitor I; Cayman #14965) [48,49,50]. Furin specifically cleaves MMP-14, which results in canonical activation of pro-MMP-2 [48,49,50]. We observed that inhibition of the MMP-2 canonical activation pathway was more prominent in control cells than in HT-29/Snail cells, in which no statistically significant decrease was observed after 24 h of incubation with the furin inhibitor (Figure 3, diagonally-striped bars; black: control, grey: HT-29/Snail). These results suggest that MMP-14 is not a master regulator of MMP-2 gelatinolytic activity in HT-29 cells overexpressing Snail. The regulation of MMP-2 activity was further investigated by using ARP101, a selective MMP-2 inhibitor [51]. We observed that inhibition of MMP-2 after 24 h of incubation with 10 µM ARP101 decreased the gelatinolytic ability of control cells and HT-29/Snail cells, however the percentage values were comparable (Figure 3, parallel-striped bars; black: control, grey: HT-29/Snail). In summary, we can assume that Snail overexpression modulates the mechanism of MMP-2 activation and additionally engages other proteases in pericellular gelatinolysis.

**Figure 4:**
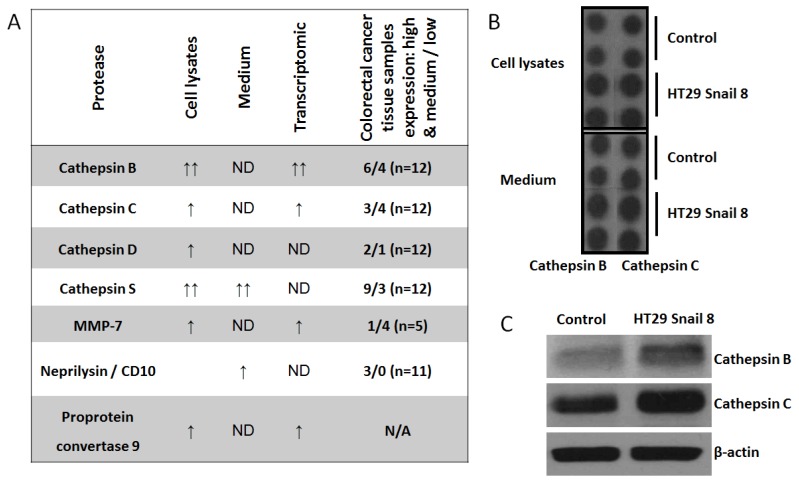
The expression of non-MMPs proteases in HT-29 cells with Snail overexpression. Cell lysates and culture medium were analyzed by using Proteome Profiler Human Protease Array (R&D System, ARY021). (**A**) Comparative analysis of transcriptomic [9] and proteome profiler data from HT-29 cells overexpressing Snail with proteomic analysis of colorectal cancer tissue samples obtained from “The Human Protein Atlas” (https://www.proteinatlas.org). (**B**) Representative picture of cathepsin B and cathepsin C expression in proteome profiler dot technique analysis. Full proteome profiling was shown in Figure S2 (**C**) Western blot analysis of cathepsin B and cathepsin C. ND—not detected; N/A—not available.

Given the above results, we decided to screen our invasive colorectal cancer model [9] for other potential invadosome-related proteases. In this study, we used a Proteome Profiler Human Protease Array Kit (ARY021, R&D) to analyze cell lysates and culture medium from HT-29 cells overexpressing Snail. We noticed elevated levels of cathepsin B, cathepsin C, cathepsin D, cathepsin S, MMP-7, and proprotein convertase-9 in the cell lysates, and in the culture medium, cathepsin S and neprilysin levels were elevated (Figure 4A and Figure S2). Then, we compared the obtained results with our previous transcriptomic data from HT-29/Snail cells [9] and data from a proteomic analysis of colorectal cancer tissue samples from the Human Protein Atlas (https://www.proteinatlas.org) [36,37] (Figure 4A). For further analysis, we selected cathepsin B (CTSB) and cathepsin C (CTSC), because we detected their transcript upregulation in global transcript analysis [9] (Figure 4A); we confirmed their higher protein levels (Figure 4B) in Snail-overexpressing HT-29 cells through western blotting (Figure 4C). Interestingly, we observed higher transcript and protein levels for cathepsin B than for cathepsin C in HT-29/Snail cells (Figure 4C). According to the Human Protein Atlas, these cathepsins were identified in tissue samples from CRC patients (Figure 4A). CTSB failed to be detected in only 2 out of 12 CRC tissue samples, whereas high or medium upregulation was observed in 6/12 samples, and low upregulation was observed in 4/12 samples. Furthermore, in the case of rectum adenocarcinoma, increased CTSB levels decreased the five-year survival rate to 0% compared to 56% for low CTSB protein levels, as observed by Kaplan-Meier analysis (https://www.proteinatlas.org). CTSC was detected in 7 out of 12 CRC patient tissue samples; however, high or medium upregulation was observed in only 3 CRC patient tissue samples.

**Figure 5:**
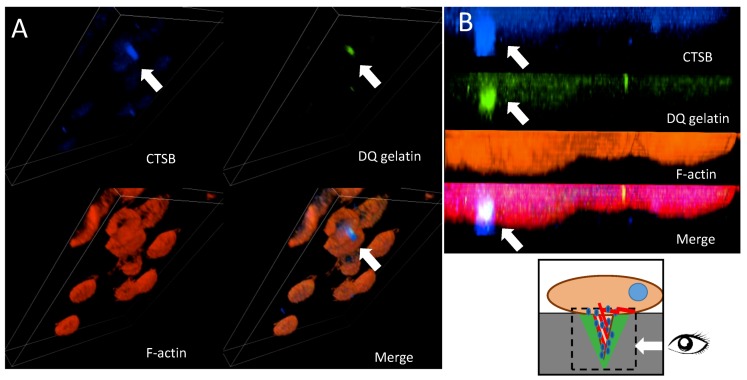
Cellular localization of cathepsin B in HT-29 cells with Snail overexpression. (**A**) 3D Z stack of HT-29 cells overexpressing Snail. Cathepsin B was visualized by confocal microscopy. Arrows indicate accumulation of cathepsin B (blue) in the cylindrical invadosome structures on ventral side of the cells visualized as F-actin staining (red), ECM degradation was visualized by fluorescence from digested DQ-gelatine (green). (**B**) Zoom of YZ axis. Arrows point co-localization of cathepsin B and digested gelatine in invadosomes.

Cathepsins B and C have been shown to be involved in increased proteolysis of the ECM [52,53]. To validate the involvement of CTSC and CTSB in invadosome-related proteolysis, we analyzed their cellular localization through confocal microscopy analysis. We performed immunostaining with specific primary antibodies followed by secondary antibodies conjugated with fluorescent probes. To visualize cathepsin B or C localization in close proximity to ECM proteolysis mediated by invadosomes in our experiments, cells were seeded on FITC-conjugated gelatine (DQ assay). The created 3D z-stacks indicated that CTSB (blue) co-localized with formed invadosomes (red) on the ventral side of HT-29/Snail cells, forming a cylindrical structure over 6 µm in depth that mediated the degradation of gelatine (green) (Figure 5A). Furthermore, the zoom of the YZ axis enables perfect localization of invasive structure formation on the ventral side of the cells [46]; thus, the cylindrical structure of the formed invadosome was precisely visualized (Figure 5B). No CTSB and invadosome co-localization was observed in control cells (data not shown).

**Figure 6:**
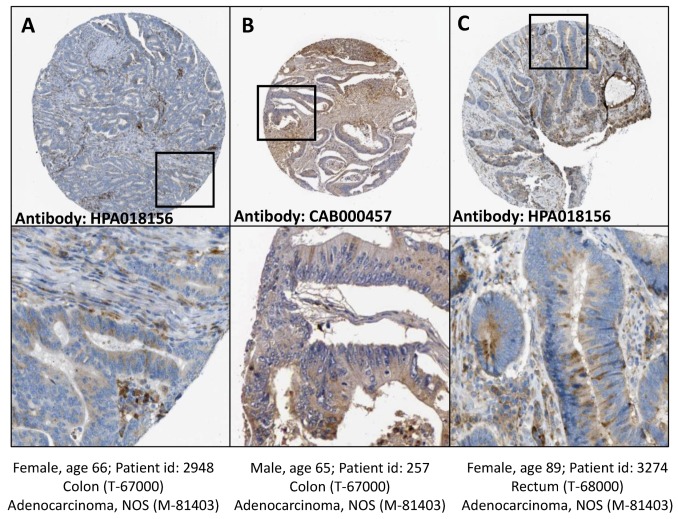
Cathepsin B detection in tissue samples from human colon and rectum adenocarcinoma. Human colon (**A**,**B**) and rectum (**C**) adenocarcinoma tissue samples, stained with anti cathepsin B antibodies HPA018156 (**A**,**C**) or CAB000457 (**B**). Cathepsin B location: cytoplasmic/membranous. Staining quality medium, staining intensity moderate. Image credits: Human Protein Atlas, www.proteinatlas.org, [36,38]. Image available at the following URL: (**A**) v18.proteinatlas.org/images/18156/41693_A_2_6.jpg; (**B**) v18.proteinatlas.org/images/457/1437_A_2_7.jpg; (**C**) v18.proteinatlas.org/images/18156/41693_A_3_2.jpg.

In contrast, CTSC accumulated near the cell-substratum interface and gelatine degradation sites but not inside the created invadosome structure (data not shown). Finally, we compared the cellular localization of cathepsin B in HT-29/Snail cells with its localization in CRC patient tissue samples. Cathepsin B has been identified as a lysosomal protease; however, in cancer cells, cathepsin B is transported to the cell surface. The data from the Human Protein Atlas (https://www.proteinatlas.org) [36,38] showed that CTSB localized in both the cytoplasm and cell membrane in colon and rectum adenocarcinoma tissues samples (Figure 6), similar to the results in our cell model (Figure 5A,B).

**Figure 7:**
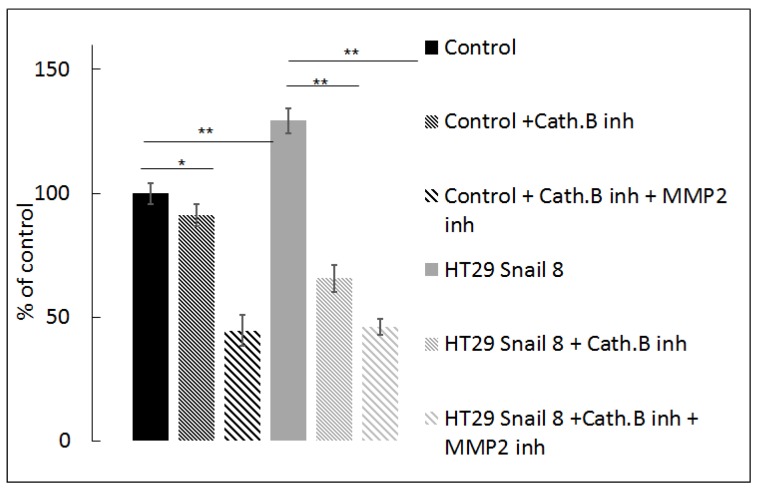
Effect of cathepsin B inhibition on gelatine degradation mediated by HT-29 cells with Snail overexpression. Cells were seeded on FITC conjugated-gelatine and incubated 24h in the presence of 100 µM CA-074Me, inhibitor of cathepsin B or combination of 100 µM CA-074Me and 10 µM ARP10, MMP-2 inhibitor. Next, cells were analyzed by measurement of increase in FITC fluorescent intensity relativized to control cells treated as 100%. ** *p* > 0.005; * *p* > 0.05.

To confirm the involvement of cathepsin B in ECM degradation, we seeded cells on FITC-conjugated gelatine and incubated them in the presence of a specific cathepsin B inhibitor, CA-074Me, suitable for whole cell experiments [54]. We observed a significant decrease (51.5%) in gelatine degradation mediated by HT-29/Snail cells after 24 h of incubation with 100 µM CA-074Me at 37 °C. In contrast, we observed a slight decrease in gelatine degradation by the control HT-29 cells (8%). Further experiments showed that a combination of 100 µM CA-074Me (cathepsin B inhibitor) and 10 µM ARP101 (MMP2 inhibitor) decreased the fluorescence intensity of FITC-conjugated gelatine to less than 50% of the control levels, both in Snail-overexpressing and control cells (Figure 7). Notably, inhibition of MMP-2 activity by ARP101 alone in HT-29/Snail cells resulted in decreased proteolysis, which was not as noticeable as that shown in Figure 4. Data analysis using Linear Interaction Effect model and Bliss Independence model based on Berenbaum’s equation [40,55,56] indicated that the combination of cathepsin B and MMP-2 inhibitors exerted a synergistic effect for gelatinolytic inhibition, suggesting the involvement of cathepsin B in gelatine degradation and/or MMP-2 activation.

**Figure 8:**
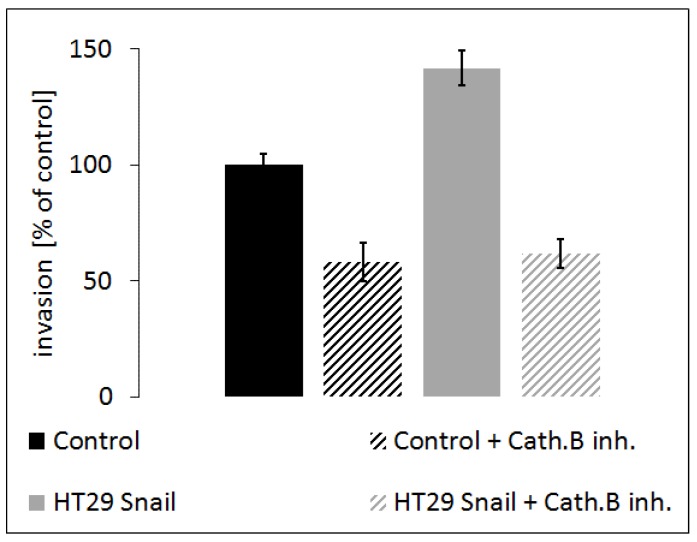
Effect of cathepsin B inhibition on invasion of HT-29 cells with Snail overexpression. Cells incubated for 24 h with cathepsin B inhibitor, CA-074ME (100 µM), or untreated once were seeded on Matrigel coated transwell inserts in the upper chamber in 0.1% BSA medium. Full medium in lower chamber served as chemoattractant for cell invasion. Cells were calculated after 6 h of incubation followed by hematoxylin/eosin staining. Number of control HT-29 cells that transmigrate into transwell membrane through 8 µM pores covered with Matrigel was set as 100%, next number of other cells was calculated and presented as % of control.

To further verify the above observation, we analyzed the influence of cathepsin B inhibition on HT-29/Snail cell invasion ability. We performed a transwell migration assay. Our results showed that HT-29/Snail cells exhibited higher invasiveness than control HT-29 cells (Figure 8; black bars: control, grey bars: HT-29/Snail). Furthermore, treatment with 100 µM CA-074Me (cathepsin B inhibitor) significantly reduced invasiveness in both HT-29 and HT-29/Snail cells (Figure 8 diagonally-striped bars; black: control, grey: HT-29/Snail), indicating that cathepsin B inhibition, which reduces pericellular proteolysis and ECM degradation and thus decreases cell invasion.

## 4. Discussion

Mesenchymal migration enables cancer cells to cross physical barriers surrounding tumors [57] and provides the possibility for disseminated tumor cells to extravasate into the parenchyma of distant organs, thus leading to the crossing of anatomical boundaries and the formation of secondary tumors [4,5]. The acquisition of mesenchymal cell traits by epithelial carcinoma cells is achieved through a programmed phenotypic shift called the epithelial-to-mesenchymal transition, EMT. The EMT is controlled by the transcription factor families Snail, ZEB and Twist [58,59,60]. Different combinations of EMT-related transcription factors orchestrate various manifestations of the EMT program. Many intermediate states have been reported to occur, with cells presenting both mesenchymal and epithelial markers and abilities [61]. Hence, the EMT can equip benign epithelial cells with the traits necessary for migration and invasion, EMT activation within tumor cells is considered a useful indicator of tumor aggressiveness. The EMT changes the expression of many genes; among them, metalloproteinases-2 and -9 (MMP-2 and MMP-9) have been reported to be upregulated [10] and are considered metastatic progression-related genes [4]. Interestingly, our previous published data did not confirm elevated MMP-2 expression or activity in stably transfected HT-29/Snail cells [9,34]. Nevertheless, we observed higher migration rates in HT-29/Snail cells than in control cells. The migration rate of HT-29/Snail clones also increased over time in a wound healing-like assay, and additionally, the extracellular matrix proteins, fibronectin, vitronectin and type I collagen, enhanced HT-29/Snail cells motility [9]. These results can be supported by two explanations. First, Snail upregulation in HT-29 cells resulted in incomplete phenotype conversion up to the intermediate epithelial state [9]. Second, in our previous study [34], we used a substrate zymography assay, an electrophoretic technique based on SDS-PAGE that involves substrate co-polymerization with a polyacrylamide gel and the detection of enzyme activity based on molecular weight separation, to determine MMP-2/-9 activity [62]. However, this technique measures the activity of the whole MMPs pool obtained from cell lysates or cell medium, and is not sufficient to accurately correlate MMPs activity with its cellular distribution [11,15,62,63]. It has been shown that MT1-MMP, MMP-2 and MMP-9 are part of the ‘standard equipment’ of the matrix-degrading cellular structure [64]. Thus, to estimate the MMPs activity related to the cellular invasive structure in the present study, we used in situ zymography; specifically, we used an alternative highly quenched version of this assay that uses quenched FITC-conjugated gelatine as a substrate. Since the cells were seeded on quenched gelatine in our experiments, we observed fluorescence in areas with gelatinolytic activity derived from the cellular surface. Our results showed that HT-29/Snail cells had greater gelatine-degradative ability than control cells. Furthermore, Snail overexpression upregulated the invadosome-related proteins Grb-2, Nck1/2 and cortactin. Grb-2 and Nck1/2 regulate F-actin dynamics and play crucial roles in the process of cell locomotion. Silencing Nck1/2 with siRNA and shRNA decreases the 3D migration of the breast cancer cell line MDA-MB-231 by dysregulating actin dynamics and invadosome formation [46]. Grb-2 is considered a podosome marker observed in non-malignant cells including macrophages, endothelial cells, and smooth muscle cells [65]. However, podosomes and invadopodia are now considered difficult to distinguish from one another, as invadopodia may evolve from simple podosomes and adapt or even change into podosomes in response to the changes in the 3D microenvironment [13,66,67,68]. Thus, it has recently been proposed that invadopodia, podosomes, and possibly other actin-based cellular protrusions that bind and degrade the extracellular matrix may represent a spectrum of molecularly related structures that may adapt and even be interchanged in response to the microenvironment and may be collectively called invadosomes [13,68]. Cortactin increases MMPs secretion as well as the cell surface presentation of MMP-14 [69]. Our results indicated that HT-29 cells with Snail overexpression presented clearly formed invadosome structures, visualized as the co-localization of Grb-2 or Nck1/2 and digested gelatine areas in confocal microscopy [70]. We estimated that invadosome-positive HT-29/Snail cells comprised between 17 and 25% of the whole cell population (observed in a random field of vision), while less than 7% of control cells were positive for invadosomes. Invadosomes can form in response to a variety of signaling molecules, such as VEGF, EGF, and TGFβ, and microenvironmental cues, such as substrate rigidity or density, hypoxia, and ROS. Each of these factors is considered a hallmark of aggressive tumors [71]. In our experimental model, Snail overexpression induced invadosome formation and gelatinolytic activity in a colon cancer cell line. Localization of proteases to invadosomes is required for ECM degradation and remodeling. Three classes of proteases were identified at invadosomes: matrix metalloproteases (MMPs), cathepsin cysteine proteases and urokinase-type plasminogen activator (uPA). Among these proteases, a transmembrane MMP, MMP-14 (MT1-MMP), has been shown to be a master regulator of invadosome function and to cleave and activate pro-MMP-2, which is transported to the forming invadosomes during cancer cell invasion [72,73,74]. Although the level of MMP-14 protein was noted altered in HT-29/Snail cells, as we showed in our previous study [9], we investigated the regulation of MMP-2 activity using Furin Inhibitor I [11,74]. Furin specifically cleaves and activates MMP-14 [48,49,50,71,75]. We focused on MMP-2 rather than on MMP-9 as MMP-2 is directly activated by MMP-14, whereas MMP-9 is activated by MMP-2 [76,77]. The decrease in gelatine degradation activity in HT-29/Snail cells in the presence of Furin Inhibitor I was not statistically significant; however, we observed a decrease in the gelatinolytic ability of HT-29/Snail cells in the presence of MMP-2 inhibitor. This finding suggests that other proteases are involved in the activation of MMP-2. MMP-2 is also activated by non-canonical pathways, which comprise enzymatic removal of pro-domains mediated by active proteolytic enzymes, e.g., MMP-2 itself or cathepsins [54]. Our data suggest that cathepsin B is involved in the gelatinolytic activity of HT-29/Snail cells. Our previous transcriptomic analysis [9] and present proteomic analysis showed that cathepsin B was upregulated in HT-29/Snail cells. Interestingly, overexpression of cathepsin B has been observed in various malignancies. Cathepsin B is involved in the EMT and is also regulated by various EMT-related factors [78]. Additionally, cathepsin B targets the cell adhesion protein E-cadherin [24], an epithelial marker responsible for cell-cell junctions [79,80]. The vast influence of cathepsin B on various aspects of tumor metastasis makes it an attractive target for cancer therapy [21,24]. Cathepsin B has been identified as a lysosomal protease that has additional extra lysosomal functions. In cancer cells, cathepsin B is transported to the cell surface, where it localizes and facilitates the ECM destruction process, orchestrating the protease cascade and directing it towards the invasive fronts of metastatic cells [81]. Cathepsin B, along with uPA, is an efficient activator of MMPs, specifically MMP-2, on the cell surface [82]. Cathepsins are active in acidic conditions; therefore, cancer cells develop numerous mechanisms of pH regulation, including electrogenic vacuolar-type H^+^-ATPases (V-ATPases) and Na^+^/H^+^ exchangers (NHEs), which are located in invadosomes and are directly involved in their function [31]. Proton delivery mediated by NHEs on the leading edge of melanoma cancer cells increases their migration rate and 3D invasion [83,84]. In HT-29/Snail cells, we observed the accumulation of cathepsin B in close proximity to the gelatinolytic areas mediated by invadosomes in a manner comparable to that observed in macrophages and v-Src fibroblasts that present mesenchymal migration [28,29]. Thus, we conclude that cathepsin B activity and invadosome localization in HT-29/Snail cells is related to a mesenchymal phenotype and correlates with mesenchymal migration in colon cancer. According to the Human Protein Atlas (https://www.proteinatlas.org), cathepsin B is highly detected in colon and rectum glandular cells but not in colon or rectum endothelial cells. Proteomic screening of tissue samples from colorectal cancer [85] patients proved that cathepsin B was significantly upregulated in CRC tissue samples. Nevertheless, in colorectal cancer tissues of less-advanced stages, cathepsin B is predominantly expressed by macrophages at the leading edge of invading tumors [85]. Since the mid-1980s, tumors have often been compared to wounds that do not heal, as they share many molecular similarities with wounds in the healing process, including neovascularization and ECM remodeling [86]. Tumor-associated macrophages (TAMs) and cancer-associated fibroblasts (CAFs) contribute to all stages of cancer development [87]. In particular, they increase tumor invasion and metastasis, promoting the EMT and the mesenchymal phenotype in cancer cells [88]. According to our findings, Snail-overexpressing HT-29 cells that imitate to have phenotype of colon cancer cells in the early stage of the EMT showed an increase in the expression and pericellular activity of cathepsin B. It appears that the pericellular localization of cathepsin B, as observed in cancer cells, plays a key role in its function. A localized approach to targeting the expression or activity of cathepsin B would prevent global suppression and the production of unintended effects. However, inhibition of total pericellular proteolysis (by inhibiting MMPs, cathepsins, uPA, etc.) attenuates mesenchymal migration, often leading to the mesenchymal-to-amoeboid transition (MAT) in which cells obtain flexible, “pathfinding” amoeba-like characteristics, as observed for HT-1080 fibrosarcoma and MDA-MB-231 carcinoma cells [89].

## 5. Conclusions

Cancer cell invasion and metastasis are very complicated and are not yet fully understood. The old paradigm states that the most important proteolytic enzymes involved in ECM remodeling and thus in breaching physiological barriers are the matrix metalloproteinases MMP-14, MMP-2 and MMP-9. However, no MMP inhibitor has proven to be clinically useful and passed clinical trials, thus leading to the assumption that many other proteases are involved in cancer cell migration. Recently, cathepsins observed in invadosome structures were proposed to be responsible for the activation of MMP zymogens as well as for the actual degradation of extracellular matrix components and junction proteins. Our data show that cathepsin B is upregulated in Snail –overexpressing HT29 cells and that it accumulates in invadosomes that accompany the mesenchymal migration ability gained when HT 29 cells shift towards a mesenchymal phenotype. Furthermore, inhibition of cathepsin activity by CA-074Me decreased the proteolytic and invasion ability of these cells. Thus, we hypothesize that the pericellular activity of cathepsin B controls ECM proteolysis related to mesenchymal migration in colon cancer cells at early stages of the EMT.

## Supplementary Materials

The following are available online at https://www.mdpi.com/2073-4409/8/3/203/s1, Figure S1: Invadosomes structures in HT-29 control cells, Figure S2: The representative image of Proteome Profiler Human Protease Array Kit (R & D System, ARY021) of medium or cell lysates from HT-29 control cells vrs. HT-29/Snail.
