# Supplementary material for: Cathepsin B Is Upregulated and Mediates ECM Degradation in Colon Adenocarcinoma HT29 Cells Overexpressing Snail
Source: Cells. 2019 Feb 27;8(3):203. doi: 10.3390/cells8030203 (PMC6468499; doi:10.3390/cells8030203)
Supplement: Supplementary file 1 [file cells-08-00203-s001.pdf]

A

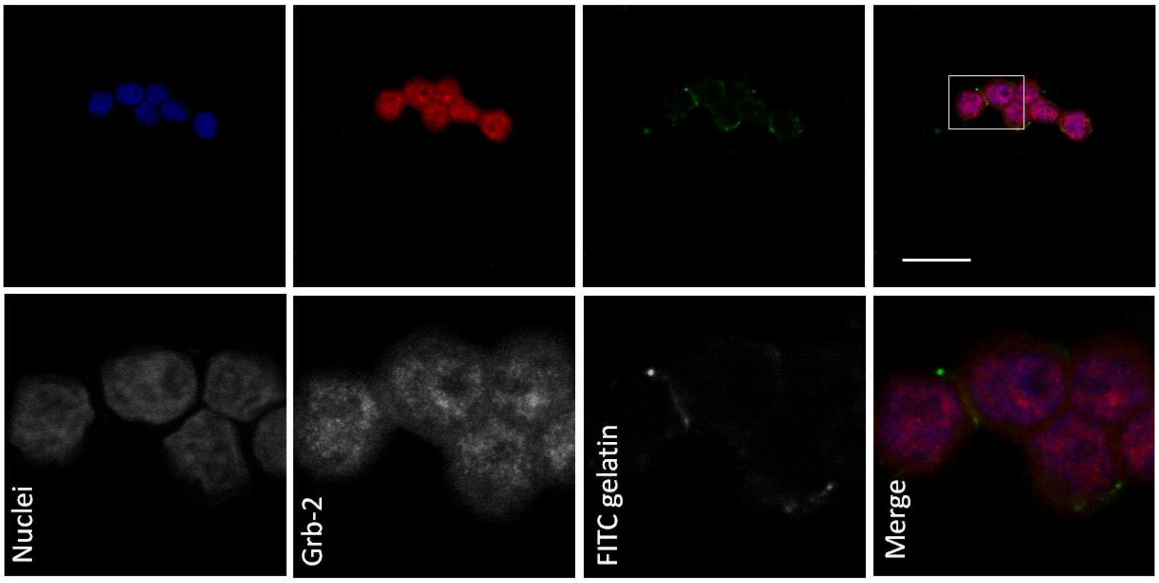

B

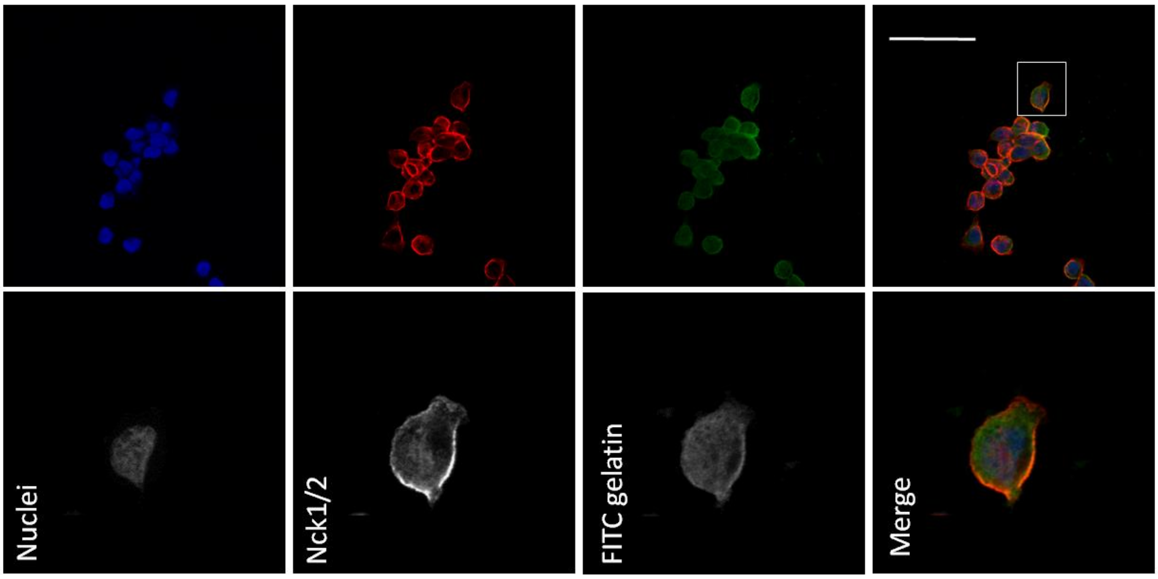

S.1. Invadosomes structures in HT-29 control cells. Cellular localization of invadosome related protein Grb-2 (A), no accumulation of Grb-2 (red) with FITC-gelatin degradation (green). Nck1/2 co-localization with gelatin degradation observed on focal – XY axis (B)

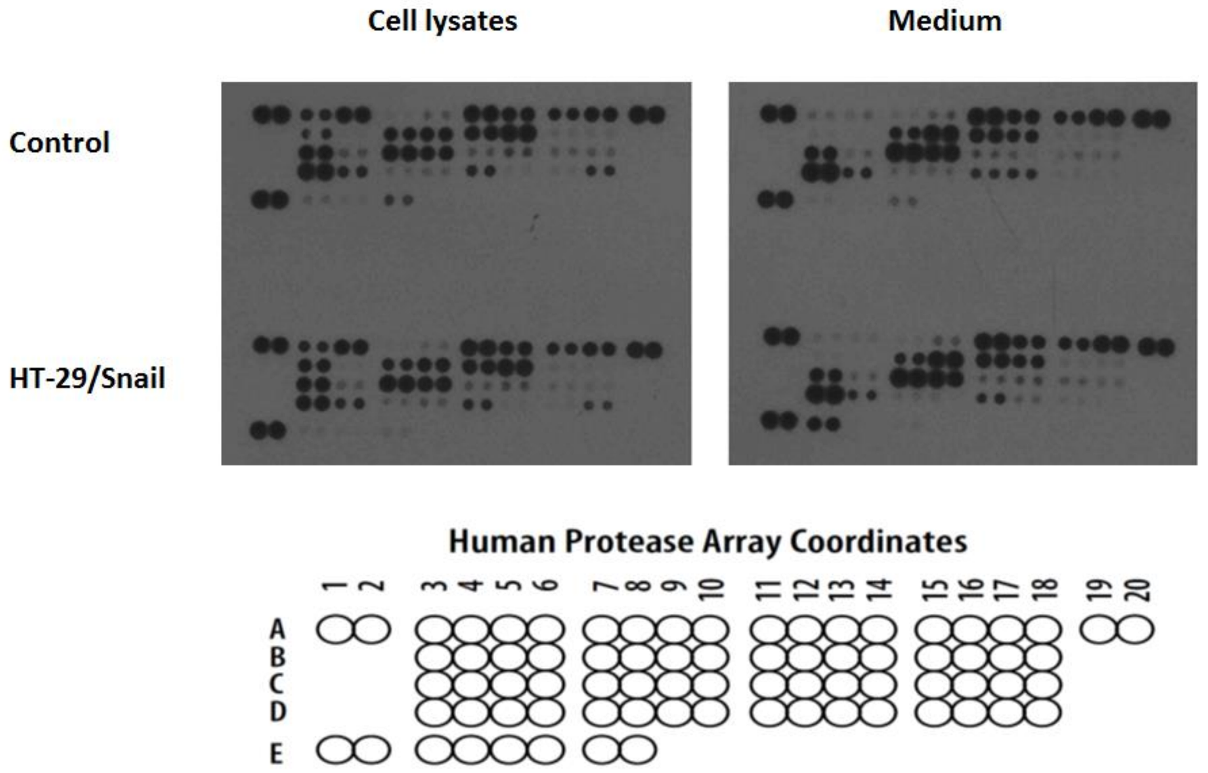

S.2. The representative image of Proteome Profiler Human Protease Array Kit (R&D System, ARY021) of medium or cell lysates from HT-29 control cells vrs. HT-29/Snail. A1, A2 Reference Spots; **A3, A4** ADAM8 Ectodomain MS2, CD156a; **A5, A6** ADAM9 Ectodomain MDC9, meltrin γ; **A7, A8** ADAMTS1 Proform METH1; **A9, A10** ADAMTS13 Active von Willebrand factor-cleaving protease; **A11, A12** Cathepsin A Proform & Active CTSA, Lysosomal Carboxypeptidase A; **A13, A14** Cathepsin B Proform CTSB, APPS, CPSB; **A15, A16** Cathepsin C Proform & Active CTSC; **A17, A18** Cathepsin D Proform & Active CTSD, CPSD; **A19, A20** Reference Spots; **B3, B4** Cathepsin E Proform & Active CTSE, CATE; **B5, B6** Cathepsin L Proform & Active CTSL, CATL, MEP, Cathepsin L1; **B7, B8** Cathepsin S Proform & Active CTSS; **B9, B10** Cathepsin V Proform & Active CTSV, CTSL2, CTSU, Cathepsin L2; **B11, B12** Cathepsin X/Z/P Proform & Active CTSX, CTSZ; **B13, B14** DPPIV/CD26 Ectodomain ADABP, ADCP2; **B15, B16** Kallikrein 3/PSA Proform & Active KLK3, hK3, KLK2A1; **B17, B18** Kallikrein 5 Proform & Active KLK5, SCTE, KLKL2; **C3, C4** Kallikrein 6 Proform & Active KLK6; **C5, C6** Kallikrein 7 Proform & Active KLK7, SCCE, PRSS6, hK7; **C7, C8** Kallikrein 10 Proform & Active KLK10, NES1, PRSSL1; **C9, C10** Kallikrein 11 Proform & Active KLK11, TLSP, PRSS20; **C11, C12** Kallikrein 13 Proform & Active KLK13, KLKL4; **C13, C14** MMP-1 Proform & Active Collagenase 1, Interstitial Collagenase; **C15, C16** MMP-2 Proform & Active Gelatinase A; **C17, C18** MMP-3 Proform & Active Stromelysin-1; **D3, D4** MMP-7 Proform & Active Matrilysin, PUMP 1; **D5, D6** MMP-8 Proform & Active Collagenase 2, Neutrophil Collagenase; **D7, D8** MMP-9 Proform & Active Gelatinase B, CLG4B, GELB; **D9, D10** MMP-12 Proform & Active Macrophage Elastase; **D11, D12** MMP-13 Proform Collagenase 3; **D13, D14** Neprilysin/CD10 Ectodomain MME, NEP, CALLA; **D15, D16** Presenilin-1 N-Terminal Fragment PSEN1, AD3, PS-1; **D17, D18** Proprotein Convertase 9 Active PC9, NARC1; **E1, E2** Reference Spots; **E3, E4** Proteinase 3 Active PRTN3, Myeloblastin; **E5, E6** uPA/Urokinase Proform & Active Urokinase-type Plasminogen Activator, PLAU; **E7, E8** Negative Control
